# Right Isomerism of the Brain in Inversus Viscerum Mutant Mice

**DOI:** 10.1371/journal.pone.0001945

**Published:** 2008-04-16

**Authors:** Ryosuke Kawakami, Alice Dobi, Ryuichi Shigemoto, Isao Ito

**Affiliations:** 1 Department of Biology, Faculty of Sciences, Kyushu University, Fukuoka, Japan; 2 Division of Cerebral Structure, National Institute for Physiological Sciences, School of Life Science, The Graduate University for Advanced Studies, Okazaki, Japan; 3 Solution-Oriented Research for Science and Technology, Japan Science and Technology Agency, Kawaguchi, Japan; Columbia University, United States of America

## Abstract

Left-right (L-R) asymmetry is a fundamental feature of higher-order neural function. However, the molecular basis of brain asymmetry remains unclear. We recently reported L-R asymmetry of hippocampal circuitry caused by differential allocation of *N*-methyl-D-aspartate receptor (NMDAR) subunit GluRε2 (NR2B) in hippocampal synapses. Using electrophysiology and immunocytochemistry, here we analyzed the hippocampal circuitry of the *inversus viscerum* (*iv*) mouse that has a randomized laterality of internal organs. The *iv* mouse hippocampus lacks L-R asymmetry, it exhibits right isomerism in the synaptic distribution of the ε2 subunit, irrespective of the laterality of visceral organs. This independent right isomerism of the hippocampus is the first evidence that a distinct mechanism downstream of the *iv* mutation generates brain asymmetry.

## Introduction

Previously we demonstrated that the synaptic distribution of NMDAR ε2 subunits in the adult mouse hippocampus is asymmetrical between apical and basal dendrites of individual neurons and between the left and right hemispheres [1], [2]. These asymmetrical allocations of ε2 subunits affect the properties of NMDARs in hippocampal synapses and generate two populations of synapses. The NMDAR-mediated excitatory postsynaptic currents (NMDA EPSCs) of the ‘ε2-dominant’ population are highly sensitive to Ro 25-6981, a ε2 subunit–selective antagonist [3]–[5]. The plasticity of these synapses develops rather early. The other population is ‘ε2-nondominant’. The NMDA EPSCs of these synapses are less sensitive to low concentrations of Ro 25-6981 and the plasticity of these synapses develops slowly. These two populations of synapses are distributed asymmetrically between the left and right hippocampus. These findings represent the first example of L-R asymmetries in synaptic composition and the function of neuronal networks within the brain.

The precise specification of L-R asymmetry is essential for vertebrate development. The molecular mechanisms that generate asymmetrical patterning are well understood for the internal organs [6]–[8] but not for the brain. To investigate the molecular basis of the formation of brain asymmetry, we examined the asymmetry of the distribution of ε2 subunits in the *iv* mouse [9]. *Iv* is a spontaneous mouse mutant that possesses a mutation in the gene encoding the motor protein, *Left-right dynein* (*Lrd*) [10]. In embryos 7.5 days postcoitum, the leftward nodal flow generated by the rotation of cilia initiates the L-R axis determination process [11], [12]. However, in *iv* homozygous (*iv*/*iv*) embryos, the nodal cilia are immotile and fail to produce constant leftward flow, resulting in a randomized laterality of visceral organs [13]–[15]. Fifty percent of *iv*/*iv* mice exhibit reversed asymmetry (*situs inversus)*, whereas the rest are normal (*situs solitus*).

Here, we report that the *iv* mouse hippocampus exhibits right isomerism of the synaptic distribution of the ε2 subunit. This laterality defect of the hippocampus is independent of the laterality of visceral organs. Therefore, the mechanisms responsible for the specification of L-R asymmetry differ between visceral organs and the brain.

## Results

### Properties of NMDA EPSCs of hippocampal CA1 pyramidal neurons

**Figure 1:**
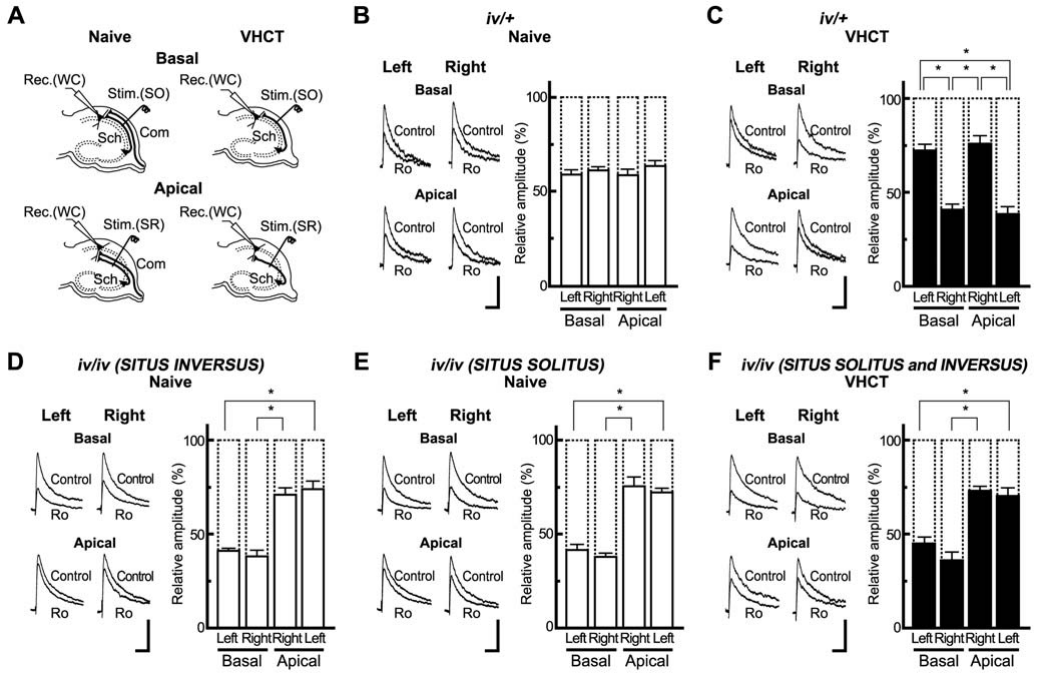
L-R laterality defects in the hippocampal circuitry of *iv/iv* mice. (A) A schematic representation of synaptic inputs onto the basal and apical dendrites of CA1 pyramidal cells and the positioning of electrodes. In slices from naive and VHCT mice, electrical stimulation was applied at the *stratum oriens* [Stim.(SO)] or *stratum radiatum* [Stim.(SR)] of area CA1. Whole-cell recordings [Rec.(WC)] were made from CA1 pyramidal cells. Basal and Apical represent recordings from basal and apical synapses, respectively. Sch, schaffer collateral fibers; Com, commissural fibers. (B to F) Inhibitory effects of Ro 25-6981 on NMDA EPSCs from CA1 pyramidal neurons. Sample superimposed traces indicate NMDA EPSCs recorded in the absence (Control) and presence of Ro 25-6981 (Ro, 0.6 µM). The levels of inhibition were maximal after exposure to Ro 25-6981 for 50 to 60 min. Left and Right indicate recordings from left and right hippocampal slices, respectively. Each trace is the average of five consecutive recordings. Scale bars, 25 pA (vertical) and 100 ms (horizontal). Relative amplitudes of NMDA EPSCs in the presence of Ro 25-6981 are expressed as percentages of control responses. Error bars represent s.e.m. (*n = *7 each, *P<0.01, absence of an asterisk indicates *P*>0.05).

First we examined properties of NMDARs in mice heterozygous for *iv* (*iv/+*). The nodal cilia rotate as rapidly in these heterozygotes as in wild-type mice (WT) [15] and the laterality of visceral organs develops normally [9]. To measure NMDA EPSCs, whole-cell recordings were made from CA1 pyramidal neurons in the presence of 6,7-dinitroquinoxaline-2,3-dione (DNQX, 20 µM) and bicuculline (30 µM) at a holding potential of +10 mV [1]. To discriminate between excitatory synapses on the apical and basal dendrites of CA1 pyramidal neurons, NMDA EPSCs were independently elicited by electrical stimuli applied either at the *stratum radiatum* or at the *stratum oriens* of area CA1 (Figure 1A). In naïve *iv/+* mice, Ro 25-6981 (0.6 µM) reduced peak amplitude of NMDA EPSCs to a similar extent in the left and right hippocampus in both the basal and apical dendrites of CA1 pyramidal neurons (Basal; left, 58.7±3% of control, n = 7 from 7 animals; right, 61±2% of control, n = 7 from 7 animals; Apical; right, 64±3% of control, n = 7 from 7 animals; left, 59±3% of control, n = 7 from 7 animals)(Figure 1B).

Next we examined hippocampal slices prepared from mice in which the ventral hippocampal commissure had been transected (VHCT). These mice lack commissural fibers [1]. Five days after VHCT, Ro 25-6981 sensitivity of CA1 pyramidal neurons was asymmetrical. In basal synapses, Ro 25-6981 diminished NMDA EPSCs more in right hippocampal slices than in left hippocampal slices (left, 72±3% of control, n = 7 from 7 animals; right, 41±3% of control, n = 7 from 7 animals; *P*<0.01, *t*-test)(Basal, Figure 1C). Conversely, in apical synapses, hippocampal slices from the left exhibited a greater sensitivity to Ro 25-6981 compared to the right (right, 76±4% of control, n = 7 from 7 animals; left, 38±4% of control, n = 7 from 7 animals; *P*<0.01)(Apical, Figure 1C). These results in *iv/+* hippocampus are similar to those previously reported for WT hippocampus [1]. Thus, the laterality of hippocampal circuitry develops normally in *iv/+* mice.

Next, we examined naïve *iv/iv* mice with the *situs inversus* phenotype. The sensitivity of NMDA EPSCs to Ro 25-6981 was comparable between the left and right hippocampus for both basal and apical synapses (Figure 1D). However, basal synapses exhibited a higher sensitivity to Ro 25-6981 than apical ones (Left: basal, 41±2% of control, n = 7 from 7 animals; apical, 74±4% of control, n = 7 from 7 animals, *P*<0.01; Right: basal, 38±3% of control, n = 7 from 7 animals; apical, 71±4% of control, n = 7 from 7 animals; *P*<0.01) (Figure 1D). Almost identical results were obtained with *iv/iv* mice with *situs solitus* (Left: basal, 42±3% of control, n = 7 from 7 animals; apical, 72±2% of control, n = 7 from 7 animals, *P*<0.01; Right: basal, 38±2% of control, n = 7 from 7 animals; apical, 75±5% of control, n = 7 from 7 animals; *P*<0.01) (Figure 1E). In addition, we obtained similar results with VHCT *iv/iv* mice and naïve *iv/iv* mice (left: basal, 44±4% of control, n = 7 from 7 animals; apical, 70±5% of control, n = 7 from 7 animals, *P*<0.01; right: basal, 36±4% of control, n = 7 from 7 animals; apical, 73±2% of control, n = 7 from 7 animals, *P*<0.01) (Figure 1F). In this experiment, the results obtained from animals with *situs solitus* and *situs inversus* (n = 3 to 4 each) were combined because there were no significant differences between the two groups. The sensitivity of basal and apical synapses in *iv/iv* mice to Ro 25-6981 was indistinguishable from that of ε2-dominant and nondominant synapses in VHCT *iv/+* mice, respectively.

### Development of NMDAR-dependent synaptic plasticity

**Figure 2:**
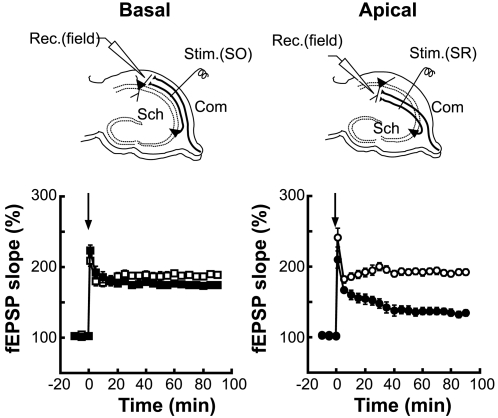
Developmental asymmetry of LTP at hippocampal CA1 synapses. Schematic diagrams of the arrangement of electrodes for extracellular recording. To activate basal (Basal) or apical dendritic synapses (Apical), a stimulating electrode was placed at the *stratum oriens* [Stim.(SO)] or *stratum radiatum* [Stim.(SR)] of area CA1, respectively. fEPSPs were recorded with an extracellular electrode [Rec.(field)]. LTP was induced with tetanic stimulation at time 0 (arrow). Open and filled symbols represent 7- to 9-week-old mice and postnatal 9- to11-day-old mice, respectively. Square and circle symbols indicate recordings from basal and apical dendritic synapses, respectively. Error bars represent s.e.m. (*n = *7 to 9).

To characterize the features of synapses in *iv/iv* mice that show the high and low sensitivity to Ro 25-6981, we analyzed the development of long-term potentiation (LTP) of field excitatory postsynaptic potentials (fEPSPs). We measured the amplitude of LTP in 7- to 9-week-old adult mice and compared it with LTP in 9- to 11-day-old pups. The ε2 subunit is the major ε subunit in the hippocampus at early postnatal ages [16], [17]. We examined slices from the left hippocampus in this experiment. fEPSPs were recorded with an extracellular electrode placed either in the *stratum oriens* or in the *stratum radiatum* of area CA1 (Figure 2). In basal synapses, the amplitudes of hippocampal LTP were similar between pups and adults (pups, 174±3%, n = 7 from 7 animals; adults, 189±3%, n = 9 from 9 animals, *P*>0.05) (Basal, Figure 2). In contrast, in apical synapses, the amplitudes of hippocampal LTP were greater in adults than pups (pups, 132±5%, n = 7 from 7 animals; adults, 192±4%; n = 9 from 9 animals, *P*<0.01) (Apical, Figure 2). Thus, LTP of Ro 25-6981 high-sensitivity synapses developed earlier than LTP in Ro 25-6981 low-sensitivity synapses. These differences in synaptic function are likely the consequence of the differential distribution of ε2 subunits at these two populations of synapses.

### Immunocytochemical analysis of the synaptic distribution of the ε2 subunit

**Figure 3:**
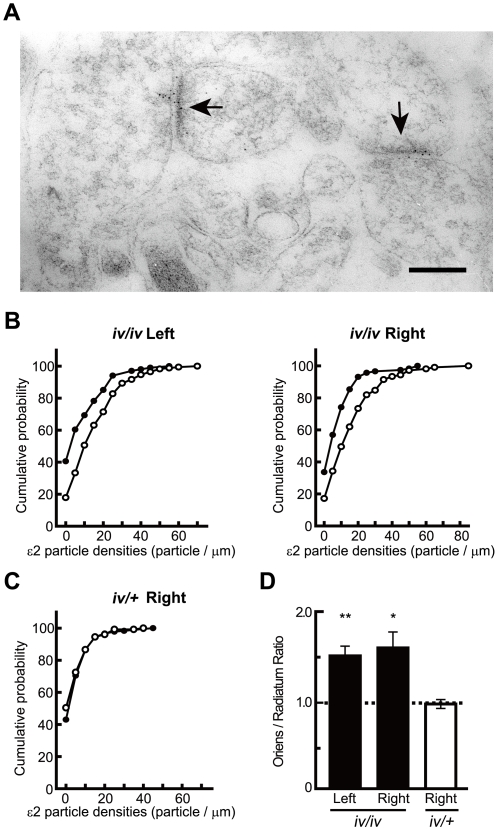
Postembedding immunogold labeling for ε2 subunits in Sch/Com-CA1 synapses in *iv* mice. (A) Immunogold particles (5 nm) for ε2 subunit are concentrated at synapses (arrows) on spines of pyramidal neurons. Scale bar, 200 nm. (B & C) Cumulative probability curves of ε2 particle density in pyramidal cell spine synapses in the *stratum oriens* (closed circles) and in the *stratum radiatum* (open circles) in *iv*/*iv* and *iv*/+ mice at postnatal day 9. This distribution is significantly different (Kolmogorov–Smirnov test, p<0.05) between the *stratum oriens* and the *stratum radiatum* in *iv*/*iv* but not in *iv*/+ mice. (D) Averaged ratios (*stratum oriens*/*stratum radiatum*) of ε2 particle density at pyramidal synapses (n = 3 each, t-test *P = 0.08 **P = 0.04, absence of an asterisk indicates *P*>0.10).

To investigate directly the synaptic distribution of ε2 subunits, we compared postembedding immunogold labeling for ε2 subunit between the apical and basal dendrites of CA1 pyramidal neurons. We examined 9-day-old mice in this experiment because the expression of ε1 subunits in the hippocampus at this age is absent or very low, whereas the ε2 subunit is already expressed at high levels [16], [17]. Therefore, at this stage of development, we consider the number of functional NMDARs to be proportional to the number of ε2 subunits at each synapse. Thus, immunogold labeling reflects both the ε2 subunit content and the number of functional NMDARs [2] (Figure 3A). The labeling density for the ε2 subunit in *iv/iv* mice was higher in the *stratum oriens* than in the *stratum radiatum* bilaterally (*iv/iv*, left, oriens/radiatum ratio = 1.50±0.1, n = 3 animals, *P* = 0.04; *iv/iv*, right, oriens/radiatum ratio = 1.59±0.2, n = 3 animals, *P* = 0.08) (Figure 3B & D), whereas no significant difference was detected in *iv/+* mice (*iv*/+, right, oriens/radiatum ratio = 0.97±0.1, n = 3 animals, *P*>0.10) (Figure 3C & D).

## Discussion

### Two distinct populations of synapses in the *iv* mouse hippocampus

The adult WT hippocampus contains two populations of synapses with distinct distributions of ε2 subunits [1], [2]. The NMDA EPSCs of the ‘ε2-dominant’ population are highly sensitive to Ro 25-6981 and LTP of these synapses develops rather early. In contrast, the NMDA EPSCs of the ‘ε2-nondominant’ population are less sensitive to Ro 25-6981 and LTP of these synapses develops slowly. In this study, we discover that the *iv* mouse hippocampus also contains two separable populations of synapses on apical and basal dendrites of CA1 pyramidal neurons. These two populations of synapses have complementary properties. First, the NMDA EPSCs of basal synapses exhibit a higher sensitivity to Ro 25-6981 than those of apical synapses (Figure 1). Second, LTP of basal synapses develops earlier than that of apical synapses (Figure 2). Third, the concentration of the ε2 subunits, examined in the hippocampus of neonates, is higher in basal synapses than in apical synapses (Figure 3). The distinct Ro 25-6981 sensitivity of NMDA EPSCs in adult hippocampal synapses indicates that differential distribution of the ε2 subunit is maintained until adulthood. On the basis of these findings, we conclude that the *iv* mouse hippocampus also contains ε2-dominant and ε2-nondominant synapses and their functional properties are very similar to those in WT mice.

The developmental difference observed in LTP can be explained by the difference in the number of functional NMDA receptors at these synapses. In early postnatal animals such as 9- to 11-day-old mice, the expression of ε1 subunits in the hippocampus is absent or very low, whereas the ε2 and ζ1 subunits are already expressed at high levels [16], [17]. In this circumstance, the differential allocation of ε2 subunits produces distinct numbers of NMDA receptors in these synapses, resulting in differential ability to express synaptic plasticity [1], [2]. Therefore, LTP of the ε2-dominant basal synapses develops earlier than that of the ε2-nondominant apical synapses (Figure 2).

### Laterality defects of the *iv* mouse hippocampus

**Figure 4:**
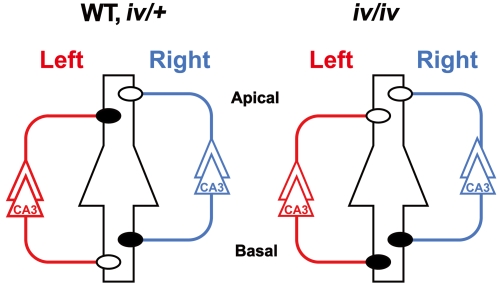
Right isomerism of the *iv*/*iv* mouse hippocampus. Left and right CA3 pyramidal neurons and their axons are colored red and blue, respectively. A postsynaptic CA1 pyramidal neuron is at the center, colored black, and it represents postsynaptic neurons in both left and right hemispheres. Closed and open circles are ε2-dominant and ε2-nondominant synapses, respectively. Apical, apical dendrites; Basal, basal dendrites.

In WT mice, the localization of ε2-doninant and ε2-nondominant synapses within hippocampal circuitry is asymmetrical (Figure 4, WT) [1], [2]. Inputs from CA3 pyramidal neurons residing in the hippocampus of the left hemisphere form ε2-dominant synapses on the apical dendrites and ε2-nondominant synapses on the basal dendrites of CA1 pyramidal neurons in both hemispheres. In contrast, inputs from CA3 pyramidal neurons in the right hippocampus form ε2-dominant synapses on the basal dendrites and ε2-nondominant synapses on the apical dendrites of CA1 pyramidal neurons. However, unlike WT hippocampus, the *iv/iv* hippocampus lacks L-R asymmetry in localization of these two populations of synapses (Figure 4, *iv/iv*). In *iv/iv* mice, synapses formed on the apical and basal dendrites of CA1 pyramidal neurons are ε2-nondominant and ε2-dominant, respectively. This localization is bilateral and independent of the laterality of visceral organs.

Even more surprisingly, this laterality defect seems to be characterized by right isomerism of the *iv/iv* hippocampus because CA3 neurons in both hemispheres form ε2-dominant and ε2-nondominant synapses on the basal and apical dendrites, respectively (Figure 4, *iv/iv*), similar to CA3 neurons in the WT right hemisphere (Figure 4, WT). Thus, it appears as if the ‘left’ hippocampus is lost and both hemispheres contain a ‘right’ hippocampus. Thus, we conclude that the *iv* mouse hippocampus exhibits right isomerism with regard to the synaptic distribution of the ε2 subunit.

### Distinct mechanisms generating L-R asymmetry of the brain

Studies of early murine development reveal that embryos are initially bilaterally symmetric. Subsequently, the symmetry-breaking process in L-R determination is initiated by the leftward nodal flow on the surface of the ventral node, so that the cells in the left side of embryo adopt properties different from the right side [6]–[8], [10]. That is, the default properties are those of the right side. Although the precise mechanisms for generating the right isomerism of the *iv*/*iv* mouse hippocampus remain elusive, one possible explanation is that the *iv*/*iv* mouse brain lacks the expression of factors that distinguish the left hemisphere and thus neurons retain the default characteristics of the ‘right’ side.

The mutation of *Lrd* may be the primary cause of the laterality defect observed in the *iv*/*iv* mouse brain. We used *iv/+* mice in control experiments and there is roughly a 25% genetic background difference between the *iv/iv* and *iv/+* mice. Therefore, experiments using congenic animals will be essential for determining whether the mutation of *Lrd* is a definite cause of this laterality defect. However, the laterality defect in the *iv*/*iv* mouse hippocampus is independent of the laterality of visceral organs, suggesting that a distinct mechanism downstream of the *iv* mutation generates brain asymmetry.

Compared to recent progress in our understanding of the laterality of visceral organs [6], [7], our knowledge about molecular asymmetry of the brain is still limited [18]–[21]. This is in part a consequence of the lack of an index for investigating brain asymmetry at the molecular level with experiments *in vitro*. Our present study demonstrates that the synaptic distribution of ε2 subunits and the properties of NMDAR-mediated synaptic functions are sensitive and quantitative indices for detecting abnormalities in the L-R asymmetry of the brain. These indices in combination with the *iv* mutant mouse that lacks L-R asymmetry are a useful model system for exploring the molecular basis of brain asymmetry.

## Materials and Methods

### VHC transection

To examine synapses made by ipsilateral Sch fibers, VHC was transected 5 days before electrophysiological recording [1], [2]. The *iv/iv* (SI/Col×C57BL/6J hybrid) and *iv/+* (produced by crossing the *iv/iv* and C57BL/6J) mice (7 to 9 W) were anesthetized with an injection of pentobarbital (60 mg/Kg, i.p.) and positioned with a stereotaxic apparatus. A small piece of a razor blade (2.5 mm wide) was glued onto a rod that was clamped onto a micromanipulator. After removing a portion of the skull (3 mm wide and 4 mm long, including the bregma), the blade was inserted to a depth of 4.0 mm at the midline to transect the VHC. To avoid damaging the sagittal sinus, the blade was initially shifted 0.5 mm to the right and inserted 0.5 mm into the cerebral cortex and was then returned to the midline position as the blade was lowered. After slowly removing the blade, a piece of skull was replaced and the scalp was closed with sutures. Animals receiving this procedure were viable for more than 3 months. All experiments were performed under the guidance of Animal Experiments in Faculty of Sciences, Kyushu University and the law (No.105) and notification (No. 6) of the government.

### Electrophysiology

Transverse hippocampal slices (450 µm thick) were cut with a vibrating microtome (VT 1000S) in ice-cold artificial cerebrospinal fluid (ACSF) (in mM: NaCl, 119; KCl, 2.5; CaCl_2_, 2.5; MgSO_4_, 1.3; NaH_2_PO_4_, 1.0; NaHCO_3_, 26; glucose, 10, saturated with 95% O_2_/5% CO_2_). Brains were fixed on an agar block, which was made by two pieces of agar (with a slope of 20°) stuck together at a right angle and mounted on the cutting stage. We lowered the left rear or right rear of the brain using the agar slopes when cutting the left or right hemisphere, respectively. Slices from a similar septotemporal level were used for experiments. Recordings were made in a submerged slice chamber perfused with ACSF at room temperature. Electrodes filled with 0.9% NaCl were used for extracellular recording. Synaptic responses were evoked at 0.1 Hz using a bipolar tungsten electrode. An LTP-inducing tetanic stimulus was given at 100 Hz for 1 s at baseline stimulus strength. The fEPSP slope was expressed as a percentage of mean slope value before the tetanic stimulation. Synaptic currents were recorded from CA1 pyramidal neurons using the blind-patch technique [22] in the whole-cell voltage-clamp mode (Axopatch 1D). A high-Mg^2+^ and Ca^2+^ (4 mM of MgSO_4_ and CaCl_2_) ACSF was used to increase membrane stability in the presence of bicuculline. Patch electrodes (3–5 MΩ) were filled with an intracellular solution (in mM: cesium gluconate, 122.5; CsCl, 17.5; HEPES buffer, 10; EGTA, 0.2; NaCl, 8; Mg-ATP, 2; Na_3_-GTP, 0.3; pH 7.2). We recorded NMDA EPSCs at +10 mV in the presence of DNQX (20 µM) and bicuculline (30 µM). We adopted a relatively low holding potential to obtain stable recordings of NMDA EPSCs throughout the experiment [1], [2]. Series resistance (10–30 MΩ) was regularly monitored during recordings. Cells were rejected if more than a 20% change occurred during the experiment. All records were filtered at 2 kHz, digitized at 4 kHz, and stored on a computer equipped with an A/D converter (Mac Lab 2e). No failures were detected in our experiments. All data were expressed as means±s.e.m. and analyzed with Student's *t*-test.

### Tissue preparation for electron microscopy

The *iv/iv* and *iv/+* mice at postnatal day 9 were anesthetized with an injection of pentobarbital (60 mg/Kg, i.p.) and transcardially perfused with 25 mM PBS pH7.4 followed by a fixative containing 4% paraformaldehyde, 0.05% glutaraldehyde and 0.5% picric acid in 0.1 M phosphate buffer (PB) pH 7.4 for 15 min. After perfusion, the brains were removed and 100 and 500 µm-thick coronal slices were alternately cut from the left and right dorsal hippocampus.

### Postembedding immunogold labeling

For postembedding labeling, the middle CA1 areas (0.5×1.0 mm) were trimmed from 500-µm-thick slices of the left and right hippocampus and cryoprotected in 10, 20, and 30% glycerol in 0.1 mM PB pH 7.4 overnight. Samples were then frozen with liquid propane (−185°C) in a cryofixation unit (EM CPC). Freeze substitution and low-temperature embedding in Lowicryl HM20 were performed as described previously [23]. Briefly, the samples were immersed in 1% uranyl acetate dissolved in anhydrous methanol (−90°C, 24 h) in a cryosubstitution unit (EM AFS). The temperature was then raised (4°C/h) from –90°C to –45°C. The samples were washed three times with anhydrous methanol and infiltrated with Lowicryl HM20 resin (Polysciences) at –45°C with a progressive increase in the ratio of resin to methanol. Polymerization was performed with ultraviolet light (360 nm) at –45°C for 24 h and 0°C for 36 h. Postembedding immunogold reaction was performed as described previously [24]. Lowicryl-embedded ultrathin sections (90-nm thickness) were picked up onto grids (nickel 400 mesh) coated with coat-quick “G” medium (Daido Sangyo). The sections were incubated in blocking solution (2% human albumin serum in TBS with 0.1% Triton X-100) for 30 min, and then in the same solution containing an anti-NR2B antibody [2] overnight at room temperature. After several washes with TBS for 30 min, the sections were incubated with 5-nm gold anti-rabbit IgG secondary antibody (British Biocell International) diluted (1∶100) in blocking solution containing polyethyleneglycol (molecular weight, 7500 kDa, 5 mg/ml) for 3 h. Sections were washed in ultrapure water, contrasted with uranyl acetate and lead citrate, and examined with a Jeol 1010 electron microscope. Mean oriens/radiatum ratios of ε2 labeling densities (n = 3 animals) were calculated by determining the oriens/radiatum ratio for each animal by dividing mean densities of immunogold particles (n = 101 to 179) in postsynaptic membrane specializations in the stratum oriens by those in the stratum radiatum.
